# Alterations of Specific Lymphocytic Subsets with Aging and Age-Related Metabolic and Cardiovascular Diseases

**DOI:** 10.3390/life10100246

**Published:** 2020-10-17

**Authors:** Ying Jen Chen, Yi Jen Liao, Van Thi Ngoc Tram, Chung Hao Lin, Kuo Chen Liao, Chao Lien Liu

**Affiliations:** 1 Division of General Internal Medicine and Geriatrics, Department of Internal Medicine, Chang Gung Memorial Hospital, Taoyuan 33305, Taiwan; ma2875@cgmh.org.tw (Y.J.C.); enzhou.lin@gmail.com (C.H.L.); kuochenliao@gmail.com (K.C.L.); 2 School of Medicine, College of Medicine, Chang Gung University, Taoyuan 33305, Taiwan; 3 School of Medical Laboratory Science and Biotechnology, College of Medical Science and Technology, Taipei Medical University, Taipei 11031, Taiwan; yjliao@tmu.edu.tw (Y.J.L.); ngoctramlabo@gmail.com (V.T.N.T.); 4 Ph.D. Program in Medical Biotechnology, College of Medical Science and Technology, Taipei Medical University, Taipei 11031, Taiwan

**Keywords:** aging, E-MDs, E-CVDs, immunosenescence, T_EM_, T_EMRA_

## Abstract

To investigate the association of immunosenescence with aged-related morbidity in the elderly, a clinical study was conducted to analyze and compare the alterations in peripheral blood (PB) T-cell subsets among young healthy (YH) controls, elderly healthy (EH) controls, and age-matched elderly patients with metabolic diseases (E-MDs), with cardiovascular diseases (E-CVDs) or with both (E-MDs/E-CVDs). The frequencies of CD3T, CD8T and invariant natural killer T (iNKT) cells were decreased in the EH, E-MD and E-CVD cohorts, indicating a decline in defense function. Although CD4T and regulatory T (Treg) cell frequencies tended to increase with aging, they were lower in patients with E-MDs and E-CVDs. Subset analyses of T-cells consistently showed the accumulation of senescent T-cell in aging and in patients with E-MDs and E-CVDs, compared with YH volunteers. These accumulated senescent T-cells were undergoing apoptosis upon stimulation due to the replicative senescence stage of T-cells. In addition, serum levels of cytokines, including interferon (IF)-γ, transforming growth factor (TGF)-β and growth differentiation factor (GDF)-15, consistently reflected alterations in T-cell subsets. This study demonstrated that T-cell subset changes with paralleled alterations in cytokines were associated with aging and age-related pathogenesis. These altered T-cell subsets and/or cytokines can potentially serve as biomarkers for the prevention, diagnosis and treatment of age-related morbidities.

## 1. Introduction

With a global increase in aging populations, many societies are facing serious challenges due to age-related diseases [1]. Aging of the immune system (known as immune aging or immunosenescence), characterized by decreased innate and adaptive immunity [2], likely contributes to this age-related morbidity [3].

Thymus, the central T-cell-generating organ responsible for T-cell generation, involutes with age. Because of thymus involution, the thymic generation of new T-cells cannot meet the replenishment demands required by the elderly [4]. After the age of 40–50 years, almost the entire T-cell supplement is derived from existing T_N_ and memory T-cells [5], with decreased frequencies of T_N_ cells, contracted T-cell receptor diversity [6,7], an increase in oligoclonal memory subsets, and the accumulation of senescent T-cells due to an insufficient homeostatic mechanism associated with aging [8]. Furthermore, Treg population, specifically naturally occurring (n)Treg cells (a key immune regulator), accumulated with advancing age [9,10,11].

Age-related changes, especially in the T-cell compartment, have been linked to the development of age-related diseases, including metabolic syndrome [12], autoimmune disorders [13], neurodegenerative disorders [14,15], heart failure [16] and cancers [17,18]. For example, the accumulation of senescent T (CD28^−^CD57^+^) cells in patients with atherosclerotic diseases as well as in those with hypertension [19,20] strongly suggested the association of immunosenescence with age-related morbidity. Moreover, aged-related cardiovascular diseases (CVDs) or metabolic diseases (MDs), a chronic inflammatory condition, are strongly associated with the increased frequency of activated T-cells and the enhancement of senescence-like features, such as reduced telomerase activity and high proportions of senescent T-cells [21]. Nevertheless, a detailed characterization of T-cell senescence, specifically T-cell replicative senescence, observed in aging and major age-related MDs and CVDs, has not previously been performed. The decreased regulatory function of Treg and impaired IL-2 signaling were found in patients with type 2 diabetes mellitus (T2D) [22]. However, the effect of aging on Treg cells and the possible association of Treg alteration with age-related pathophysiology in elderly people remain unclear.

In the current study, we investigated the interplay between T-cell subsets, including iNKT, Treg, CD4T and CD8T cells, and their detailed subpopulations, in the peripheral blood (PB) cells of elderly outpatients and age-related pathogeneses. Specifically, we analyzed the association of aging and age-related MDs and CVDs with the frequencies of various T-cell subsets and the related cytokines’ production, including transforming growth factor (TGF)-β, interferon (IFN)-γ, and growth differentiation factor (GDF)-15 levels. We showed that alterations in T-lymphocyte subsets and the accumulation of terminally differentiated (senescent) T-lymphocyte with a replicative senescence feature were significantly associated with aging and age-related MDs and CVDs. This finding allows the development of both diagnostic and therapeutic biomarkers for age-related MD and CVD, and also provides novel information for understanding the pathophysiological roles of inflammation that occur with aging in older populations.

## 2. Results

### 2.1. E-MD, E-CVD, and E-MD/CVD Groups Had Decreased CD4T Cell and Treg Cell Subsets Compared to EH

**Figure 1:**
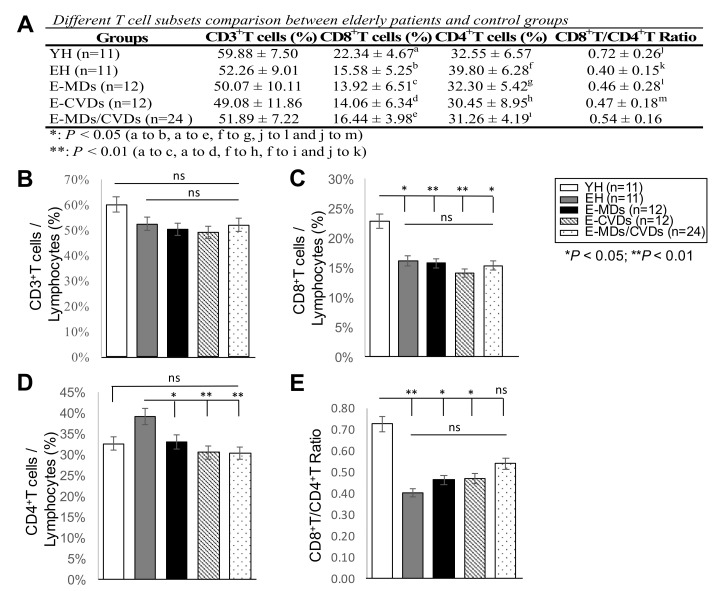
Comparison of different T-cell subsets in peripheral blood (PB) among different groups. Frequencies (%) of CD3^+^ T-cells (**A**,**B**), CD8^+^T cells (**A**,**C**), CD4^+^T cells (**A**,**D**) and the ratio of CD8^+^T to CD4^+^T (**A**,**E**) were analyzed by flow cytometry in peripheral blood (PB) from young healthy controls (YH, *n* = 11), elderly healthy controls (EH, *n* = 11), elderly patients with metabolic diseases (E-MDs, *n* = 12), elderly patients with cardiovascular diseases (E-CVDs, *n* = 12), and elderly patients with both MDs and CVDs (E-MDs/E-CVDs, *n* = 24). Data are presented as the mean ± SD for individual groups (* *p* < 0.05; ** *p* < 0.01).

**Figure 2:**
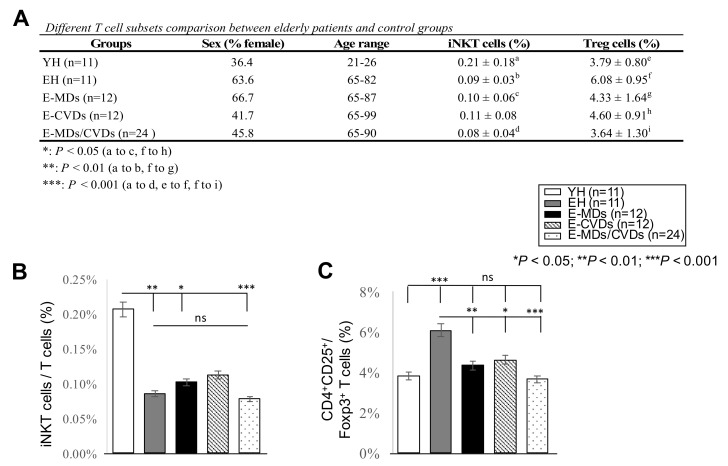
Comparison of invariant natural killer T (iNKT) and regulatory T (Treg)-cells in peripheral blood (PB) among different groups. Frequencies (%) of iNKT cells (**A**,**B**) and Treg (CD4^+^CD25^+^Foxp3^hi^) cells (**A**,**C**) were analyzed by flow cytometry in PB from young healthy controls (YH, *n* = 11), elderly healthy controls (EH, *n* = 11), elderly patients with metabolic diseases (E-MDs, *n* = 12), elderly patients with cardiovascular diseases (E-CVDs, *n* = 12), and elderly patients with both MDs and CVDs (E-MDs/E-CVDs, *n* = 24). Data are presented as the mean ± SD of individual groups (* *p* < 0.05; ** *p* < 0.01; *** *p* < 0.001).

Flow cytometry was used to analyze the frequencies of different T-cell subsets, namely CD3T, CD4T, CD8T, iNKT and Treg cells, in the PB samples of healthy elderly (EH) subjects, and compared them to those of healthy young (YH) subjects and the three elderly diseased groups (E-MDs, E-CVDs, and E-MDs/CVDs). The total number of peripheral blood mononuclear cells (PBMCs) collected through flow cytometry from individual samples was consistently 5 × 10^5^ cells; thus, each immune subset comparison between different groups was represented as a percentage (%). The results indicated that compared to YH, the EH had a decreased CD8T frequency (%, * *p* < 0.05, Figure 1), a decreased CD8T/CD4T ratio (** *p* < 0.01, Figure 1), decreased iNKT cells frequency (%, ** *p* < 0.01, Figure 2) and increased Treg frequency (%, *** *p* < 0.001, Figure 2), implying a dramatic reduction in immune defense function in aging. Interestingly, compared with EH controls, we observed significantly decreased frequencies (%) of CD4T in the E-MD, E-CVD and E-MD/E-CVD groups (%, * *p* < 0.05, ** *p* < 0.01, ** *p* < 0.01, respectively; Figure 1), indicating that in these diseased individuals, CD4T cells were more susceptible to apoptosis induction upon antigenic stimulation. Treg frequencies in the E-MD, E-CVD and E-MD/E-CVD groups also significantly decreased compared to EH (%, * *p* < 0.05, ** *p* < 0.01, *** *p* < 0.001, respectively; Figure 2), indicating an impairment of immune tolerance and/or regulation when eliciting antigenic stimulation.

We compared the frequencies of different immune cell populations, namely total white blood cells (WBCs; 10^9^/L), neutrophils, monocytes and lymphocytes in PB, among different groups by using regular complete blood counting (automatic CBC counts) (Table 1). The total number of WBCs was significantly higher in patients with E-CVDs and E-MDs/E-CVDs than in EH and YH controls (10^9^/L; * *p* < 0.05 and ** *p* < 0.01, respectively; Supplemental Figure S1). However, the frequencies of different cell populations, namely neutrophils, monocytes and lymphocytes, showed no statistically significant difference among the five groups (Table 1). We also compared correlated clinical parameters among these groups and found that the body mass index (BMI) significantly increased in the three elderly disease groups compared with EH or YH controls, for both males and female (Table 1; * *p* < 0.05, ** *p* < 0.01 and *** *p* < 0.001, respectively).

### 2.2. The Proportions of CD8 -T_N_ Cells Were Reduced Whereas CD8-T_EM_ Cells and CD8-T_EMRA_ Were Increased in Most Elderly Groups Compared to YH, Together with Increased Frequencies of CD28-CD57+CD8+ Cells

**Figure 3:**
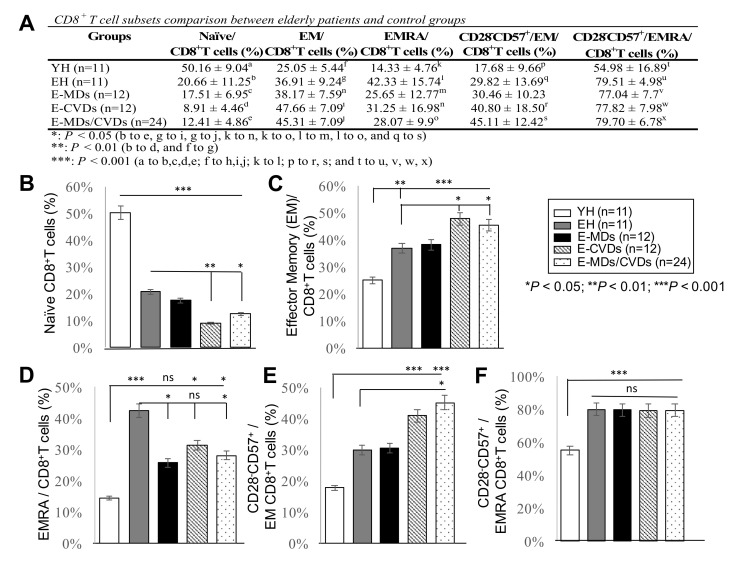
Comparison of CD8 T lymphocyte subset profiles in peripheral blood (PB) among different groups. Frequencies (%) of naïve CD8T cells (**A**,**B**), effector memory CD8T cells (**A**,**C**), EMRA-CD8T cells (**A**,**D**), CD28-CD57+CD8-T_EM_ cells (**A**,**E**), and CD28-CD57+CD8-T_EMRA_ cells (**A**,**F**) were analyzed by flow cytometry in peripheral blood from young healthy (YH; *n* = 11), elderly healthy (EH; *n* = 11), elderly with metabolic diseases (E-MDs; *n* = 12), elderly with cardiovascular diseases (E-CVDs; *n* = 12), and elderly with both E-MDs and CVDs (E-MDs/E-CVDs; *n* = 24). Data are presented as the mean ± SD of individual group comparisons (* *p* < 0.05; ** *p* < 0.01; *** *p* < 0.001).

We investigated whether aging and/or two major elderly diseases, E-MDs and E-CVDs, influenced the CD8T cell subpopulations and also determined the frequencies of different subsets of CD8T cells, including T_N_ cells, T_EM_ cells and effector memory T-cells re-expressing CD45RA (T_EMRA_), along with the loss of CD28 and the gain of CD57 (CD28^−^CD57^+^), known as the most differentiated/senescent markers [23], in both T_EM_ and T_EMRA_ subpopulations. In addition to the significant total CD8T cells reduction with aging (%; * *p* < 0.05, Figure 1), the frequencies of CD8-T_N_ in all four elderly groups were also considerably decreased compared to YH controls (%; *** *p* < 0.001). Furthermore, significant decreases in CD8-T_N_ in patients with E-CVDs or E-MDs/E-CVDs compared with EH controls (%; *** *p* < 0.001, Figure 3A,B) were also observed, indicating that thymic function may be further impaired during the onsets of MDs and CVDs in the elderly.

T_EM_ and T_EMRA_ cell subsets, which are important in CD8T cell effector function, along with specific CD28^−^CD57^+^ expression subpopulations, were also investigated. As shown in Figure 3A,C, increased frequencies of CD8-T_EM_ were observed in all four elderly groups compared to YH. The differences were even more significant in elderly diseases groups. There was a trend toward increased CD28^−^CD57^+^CD8^+^-T_EM_ in the PB of elderly groups versus YH controls (Figure 3A,E), especially in E-CVDs or E-MDs/E-CVDs groups (%; *** *p* < 0.001). The frequencies of the CD8-T_EMRA_ subset significantly increased with aging in EH compared to YH (%; *** *p* < 0.001). Interestingly, the frequencies of the CD8-T_EMRA_ subset were significantly decreased in patients with E-MDs and E-MDs/E-CVDs, compared with EH controls (%; * *p* < 0.05, respectively, Figure 3A,D), possibly due to a replicative senescence in this subset during the onset of E-MDs. We observed no differences in CD28^−^CD57^+^CD8^+^-T_EMRA_ frequencies between EH and elderly diseased volunteers, but the frequencies were significantly lower in YH controls (%; *** *p* < 0.001, Figure 3A,F).

### 2.3. The Proportions of CD4-T_N_ Cells Were Reduced While CD4-T_EM_ Cells Were Increased in Most Elderly Groups, Together with Increased Frequencies of Cd28^−^Cd57^+^Cd8^+^ Cells, While Increased Cd4-T_emra_ Cells Were Not Observed for Elderly Groups Compared to Yh

**Figure 4:**
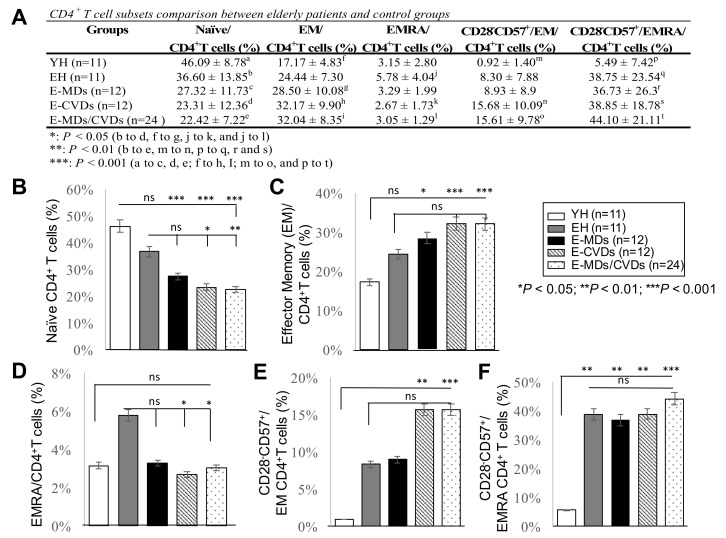
Comparison of CD4 T lymphocyte subset profiles in peripheral blood (PB) among different groups. Frequencies (%) of naïve CD4T cells (**A**,**B**), effector memory CD4T cells (**A**,**C**), EMRA-CD4T cells (**A**,**D**), CD28^+^CD57^−^CD4^+^-T_EM_ cells (**A**,**E**), and CD28^−^CD57^+^CD4^+^-T_EMRA_ cells (**A**,**F**) were analyzed by flow cytometry in peripheral blood from young healthy (YH; *n* = 11), elderly healthy (EH; *n* = 11), elderly with metabolic diseases (E-MDs; *n* = 12), elderly with cardiovascular diseases (E-CVDs; *n* = 12), and elderly with both MDs and CVDs (E-MDs/E-CVDs; *n* = 24). Data are presented as the mean ± SD of individual groups (* *p* < 0.05; ** *p* < 0.01; *** *p* < 0.001).

We examined whether the CD8T cell subpopulation alteration associated with the aging and age-related pathogenesis is also observed in CD4T cells. We therefore analyzed the frequencies of different subsets of CD4T cells in PB, including CD4-T_N_, CD4-T_EM_, CD4-T_EMRA_, and CD4^+^/CD28^−^CD57^+^ cells within T_EM_ and T_EMRA_ subpopulations (Figure 4). As expected, there was a trend toward a decreased proportion of CD4-T_N_ cells in circulation, especially for elderly MDs, CVDs, and MDs/CVDs groups compared with YH controls (%; *** *p* < 0.001, Figure 4A,B), indicating a consistent low-grade inflammatory state in aging and age-related diseases. Similar to results for CD8T subsets, CD4-T_EM_ and CD4^+^CD28^−^CD57^+^-T_EM_ cells exhibited increased frequencies in PB of age-related disease groups compared to YH controls (%; * *p* < 0.05, and *** *p* < 0.001, respectively; Figure 4A,C,E), especially for E-CVDs and E-MDs/E-CVDs groups (%; ** *p* < 0.01; Figure 4A,E), implying an accumulated senescent CD4-T_EM_ subset in E-CVD or E-MDs/E-CVDs patients.

Unexpectedly, no increase in CD4 T_EMRA_ cells in elderly groups compared to YH was detected (Figure 4D), contrary to the observation in CD8 T_EMRA_ cells. However, the frequencies of CD4^+^CD28^−^CD57^+^-T_EMRA_ in PB were significantly increased with aging and aged-related diseases (%; ** *p* < 0.01 and *** *p* < 0.001, respectively, Figure 4A,F). It is interesting to note that the frequencies of both CD8 T_EMRA_ and CD4 T_EMRA_ in the elderly diseased groups were reduced compared to EH (Figure 3D and Figure 4D), suggesting that these functionally senescent subsets with a unique migratory behavior enhanced their pathogenicity in elderly patients [24]. When we adjusted all the data for sex, we observed that the profiles of T lymphocyte subsets (CD28^−^CD57^+^/CD8^+^T_EM_, CD28^−^CD57^+^/CD4^+^T_EM_, and CD28^−^CD57^+^/CD4^+^T_EMRA_) differed by gender (Figure S5). We found a significant increase in the frequencies of CD28^−^CD57^+^/CD8^+^T_EM_ and CD28^−^CD57^+^/CD4^+^T_EM_ cells in elderly males with E-CVDs compared to females with E-CVDs (* *p* < 0.05, respectively; Figure S5A,B). The frequency (%) of CD28^−^CD57^+^/CD4^+^T_EMRA_ cells was higher in elderly males compared to elderly females (* *p* < 0.05), whereas it was lower in males with E-MDs compared to females with E-MDs (** *p* < 0.01) (Figure S5C). The clinical significance and the associated mechanisms of these observations merit further investigation.

### 2.4. Serum Levels of Ifn-γ Were Reduced with Aging While Gdf-15 Levels Were Increased in Elderly Diseases Groups Compared to Yh Controls

**Figure 5:**
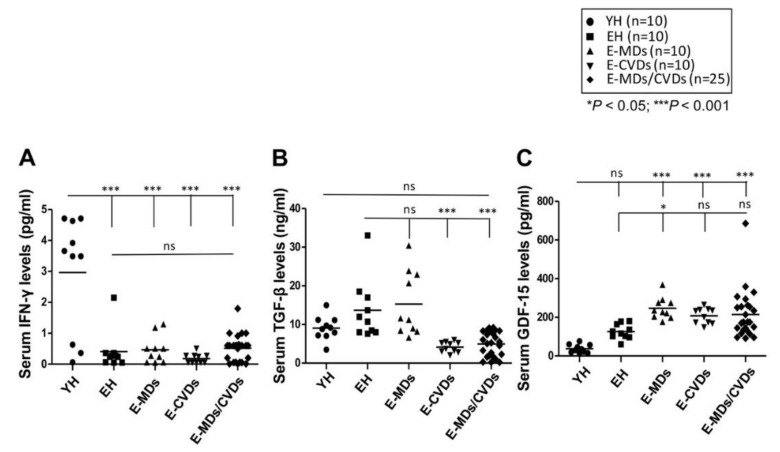
Comparison of serum cytokine levels among different groups. Serum cytokine levels, including interferon (IFN)-γ (**A**), transforming growth factor (TGF)-β (**B**) and growth differentiation factor (GDF)-15 (**C**), were investigated in young healthy (YH; *n* = 10), elderly healthy (EH; *n* = 10), elderly with metabolic diseases (E-MDs; *n* = 10), elderly with cardiovascular diseases (E-CVDs; *n* = 10), and elderly with both MDs and CVDs (E-MDs/E-CVDs; *n* = 25), measured using ELISAs. Individual data with group mean were shown for group comparison (* *p* < 0.05; ** *p* < 0.01; *** *p* < 0.001).

Evaluation of cytokine expression is becoming a useful tool for defining cell-mediated immunity [25] because it reflects immune subset alterations. To determine whether the cytokine expression levels reflect the changes in immune cell subpopulation or are associated with aging and age-related pathogeneses, we measured the serum levels of three related cytokines, namely IFN-γ, TGF-β and GDF-15, in all young and elderly individuals. We observed a significant reduction in the serum IFN-γ level in EH controls and patients with E-MDs, E-CVDs and E-MDs/E-CVDs, compared with YH controls (pg/mL; *** *p* < 0.001, Figure 5A), implying that the helper response of memory cells generated from aged naïve cells is dramatically reduced. The serum level of TGF-β, an essential cytokine secreted by Treg cells, was highest in EH controls, and the levels were significantly lower in decreased elderly groups, especially in E-CVDs and E-MDs/CVDs (ng/mL; *** *p* < 0.001, Figure 5B), in accordance with the observation of Treg proportions (Figure 2A,C). Moreover, the serum level of GDF-15 was significantly and consistently higher in patients with E-MDs, E-CVDs and E-MDs/E-CVDs (pg/mL; *** *p* < 0.001, respectively, Figure 5C), possibly due to the significant inflammation and tissue damage found in elderly MD and CVD patients.

### 2.5. T-Cell Proliferation Activities Were Relatively High in Aged People and These Proliferated T-Cells Tended to Trigger Apoptosis Following Stimulation Due to a True Senescence Phenomenon

**Figure 6:**
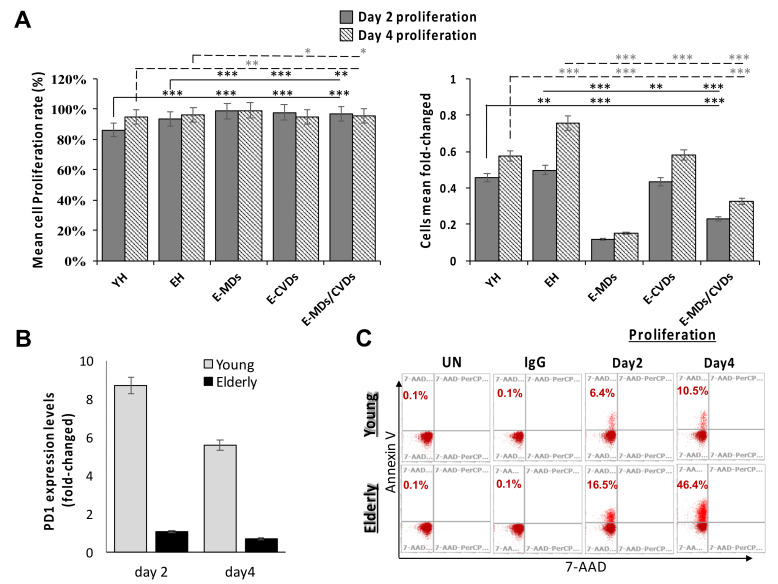
Comparison of CD3^+^ T-cell proliferation after anti-CD3/28 microbeads stimulation. CD3^+^ T-cells from each group (n = 3 per group) were stained with CFSE and cultured for 2 and 4 days following microbeads stimulation. (**A**) Comparison of mean % proliferating (CFSE^lo^) cells and cells fold-changed. (**B**) Comparison of PD1 expression levels (mean fold-changed) between young and elderly volunteers on days 2 and 4. (**C**) FACS analysis of Annexin V staining following days 2 and 4 proliferation for young and elderly volunteer groups. Data are presented as the mean ± SD in each individual group comparison (* *p* < 0.05; ** *p* < 0.01; *** *p* < 0.001).

To further investigate the T-cell functions of aged people and elderly patients with MDs and/or CVDs, we conducted a T-cell proliferation assay by using 5-(and 6)-Carboxyfluorescein diacetate succinimidyl ester (CFSE) labeling along with anti-CD3/28 microbead stimulation for 2 and 4 days. Following stimulation with anti-CD3/28 microbeads, proliferation activities on day 2 were significantly increased in the EH, MD and CVD groups (%; ** *p* < 0.01 and *** *p* < 0.001, Figure 6A left panel) compared to the YH group, whereas no such difference was observed on day 4 except for in E-MDs (Figure 6A left panel). The cell numbers significantly increased in EH compared with YH on days 2 and 4 (fold-change; ** *p* < 0.01 and *** *p* < 0.001, respectively, Figure 6A right panel), whereas cell numbers were significantly reduced in elderly patients with E-MDs, E-CVDs and E-MDs/E-CVDs, compared with EH controls, following proliferation on days 2 and 4 (fold-change; ** *p* < 0.01 and *** *p* < 0.001, Figure 6A right panel), indicating the replicative senescence of T-cells in aged-related MDs and CVDs.

To further characterize the proliferation on senescent T-cells, we measured the expression of PD1, a T-cell exhaustion marker [26], and annexin V, an apoptosis marker, following stimulation on days 2 and 4 by using a flow cytometry analysis. Because no significant differences in PD1 and annexin V levels were observed among different elderly populations, we compared the expression levels of PD1 and annexin V between young and elderly groups. As shown in Figure 6B,C, the frequencies of PD1 expression (shown by expression fold-change) were significantly higher in young people compared with the elderly population (Figure 6B), whereas the expression frequencies (shown by %) of annexin V staining were significantly increased in elderly compared with young controls following proliferation on days 2 and 4 (Figure 6C), implying T-cells with a replicative senescence that were undergoing apoptosis in elderly population.

We also compared the proliferative capacity and the expression of beta-galactosidase (a kind of SASP cytokine) and PD-1 in senescent CD4 T and CD8 T (CD28^−^CD57^+^) cells from young healthy (YH) and elderly healthy (EH) subjects. Our data showed that although the senescent CD8 (CD8^+^CD28^−^CD57^+^) T-cell numbers were twice as high in EH than in YH (data not shown), the proliferative capacity was significantly higher in EH compared to YH (similar to results shown in Figure 6A). Consistent with this, the expression levels of the replicative senescent marker beta-galactosidase activity (μU/μl) were significantly higher in EH compared to the YH following proliferation (Figure S6A). In contrast, the exhaustion marker PD1’s expression (%), which decreased with proliferation, was significantly higher in YH compared to the EH following proliferation (Figure S6A), due to a dramatic reduction in PD1 levels in EH compared to those in YH. The senescent CD4 (CD4^+^CD28^−^CD57^+^) T-cell subset seemed to behave similarly to senescent CD8T (Figure S6B), but due to the extremely limited number of senescent CD4T cells available from the peripheral blood samples of YH, a comparison of senescent CD4T cells between the YH and EH groups could not be performed. These results suggested that aged-related shifts in T-cell subsets lead to intrinsic T-cell defects, thereby altering the production of cytokines.

## 3. Discussion

We observed the significant association of changes in T-cell subsets and their related cytokine secretion with the aging process and age-related MDs and CVDs in the circulations of elderly outpatients. These T-cell subsets include conventional T-cell subsets, namely CD3T, CD4T, CD8T, Tregs and their specific subpopulations, and unconventional T-cells, namely iNKT cells, which bridge innate and adaptive immunity without restricting the classical major histocompatibility complex (MHC) molecules [27]. The frequencies of naïve CD8T and iNKT cells significantly decreased with aging; by contrast, the proportions of CD8- and CD4-T effector memory (T_EM_) cells tended to increase in aging and aged-related diseases compared to YH controls. Moreover, the frequencies of senescent CD8- and CD4-T_EMRA_ and Treg subsets were decreased in patients with E-MDs, E-CVDs and E-MDs/E-CVDs, compared to EH, while the frequencies of the most senescent subsets, CD28^−^CD57^+^ in EM (both CD4 and CD8) and EMRA (both CD4 and CD8), accumulated in elderly MD and CVD patients, suggesting that an altered T-cell with senescent features which obtained unique migratory behavior was associated with diseases in the elderly [24]. The dynamics of T-cell generation and the regulation of T-cell homeostasis are achieved by a tightly regulated balance of cell division and death, combined with the influx of newly generated naïve T-cells entering the T-cell pool [28]. One of the hallmarks of aging in the immune system is the progressive shift in the T-cell population from a predominantly naïve phenotype during youth to a mainly memory phenotype in the elderly [29]. The age-dependent memory phenotype shift is driven by exposure to a lifetime of environmental antigens, and a reduced output of naïve T-cells due to thymic involution [30]. The lower production of new naïve T-cells is balanced by the longer lifespan of naïve T-cells in the elderly to maintain a naïve T-cell pool [31], and this longer lifespan facilitates the development of age-associated defects among naïve CD4, and likely also CD8T cells, leading to an age-dependent phenotype shift in T-cells, termed immunoscenescence.

The aging process, along with MDs and CVDs, is associated with low-grade chronic inflammation and the accumulation of senescent cells in tissues [32], which is characterized by increased levels of circulating inflammatory cytokines that contribute to systemic metabolic or/and cardiovascular dysfunction. Furthermore, the altered senescent T-cell carries a senescence-associated chemokines and cytokines secretory phenotype (SASP). Our results showed that serum TGF-β levels were significantly increased in elderly patients with CVDs and MDs/CVDs compared with EH controls. Moreover, serum GDF-15, a stress-responsive cytokine of the TGF-β superfamily, produced in pathophysiological situations associated with inflammation and tissue injury (e.g., heart failure) [33], was significantly increased in elderly patients with both MDs and CVDs compared with YH and EH controls. A major factor contributing to age-related defects in immunological responses is the progressive deterioration of naïve T-cell function; therefore, aged individuals showed (1) decreased T-cell responses to neoantigens, (2) increased terminally differentiated T lymphocytes, (3) decreased T-cell receptor rearrangement, and (4) increased senescent T-cells [34,35]. In accordance with these observation, our results showed that both naïve CD4 and CD8T cells were significantly decreased in elderly people, whereas their T_EM_, T_EMRA_ and CD28^−^CD57^+^ subpopulations were significantly increased in older patients.

Senescent cells are induced by stress, which is characterized by durable growth arrest, expression of anti-proliferative molecules (e.g., telomeric foci, p16^INK4a^) and activation of damage signaling pathways (e.g., p38^MAPK^ and NF-kB), resulting in the expression of senescence-associated transcripts (e.g., β-galactosidase) [36]. However, the absolute durability of this growth arrest in vivo remains controversial. We compared the CD3T cell proliferation ability among different groups. Although we observed a higher frequency of senescent CD28^−^CD57^+^ cells with age, T-cells from the older cohort proliferated more robustly in response to anti-CD3/CD28 stimulation, suggesting that the difference in senescent cells in the age group is not sufficient to influence intrinsic T-cell responses. There are two possibilities, as follows: (1) naïve T-cells decrease and memory T-cells increase with age, which likely has a significant effect on T-cell responses, and (2) T-cells in older patients are capable of robust activation. The increased susceptibility to viral infections with age [37,38] may be due to other defects outside of T-cell activation [39]. Interestingly, the actual fold-change of cell numbers after proliferation was not in accordance with proliferation capacity; they were significantly decreased in elderly patients compared with EH and YH controls, with a significant increase in annexin V levels and a reduction in PD1 expression in elderly groups compared with YH controls following stimulation. The ability of T-cells to proliferate upon antigen stimulation (the clonal expansion) is crucial, as it dramatically increases the number of antigen-specific T-cells to aid in resolving infections. However, as T-cells replicate multiple times due to repeated stimulation with pathogens during a host’s lifetime, they further differentiate, lose their proliferation capacity, and reach the stage of replicative senescence [40]. The inability of T-cells to proliferate is partly due to the erosion of telomeres and the loss of telomerase activity [41], a phenomenon of true senescence. The mechanisms underlying this replicative senescence after robust T-cell activation in elderly people need to be further investigated.

Immunosenescence is a challenging field with many unanswered questions. Studies demonstrating alterations in adaptive immune subsets in geriatric populations leading to increased risks of E-MDs and E-CVDs remain limited. The clinical significance of these studies remains unknown, as many studies have used animal or cell culture models and have measured in vitro cytokine changes instead of real clinical outcomes. From this point of view, our investigation of alterations in T-cell subsets in clinical outpatients stratified by MD/CVD, and with standardized immunophenotyping of circulating T-cell subsets, has direct clinical relevance. The findings of the current study also suggest that continuous investigation in this direction potentially could lead to the development of biomarkers for the prevention of age-related morbidity, non-invasive diagnostics, or biomarkers for therapeutic monitoring. One limitation of the present study was the limited numbers of outpatients, especially in the EH control, since typically it is not easy for elderly people (aged >65 years) to maintain overall good health. Due to the limited numbers of patients, the impacts of complex issues, such as person-to-person variation, levels of physical activity or the socioeconomical status of subjects classified as healthy elderly cannot be addressed in the present study, and will need to be further investigated.

## 4. Materials and Methods

### 4.1. Outpatients

We enrolled 98 elderly outpatients (49 women and 49 men; age >65 years) who regularly consulted our medical center between September 2017 and January 2019, and 20 healthy elderly volunteers (EH). In total, 12 (age <40 years) healthy individuals served as young healthy (YH) controls.

### 4.2. Study Design

Elderly outpatients were divided into three groups according to the different disease categories: elderly MDs (E-MDs) only (e.g., type 2 diabetes mellitus (T2DM), hyperuricemia, and/or hyperlipidemia), elderly CVDs (E-CVDs) only (e.g., hypertension, cardiomyopathy, and/or cardiac dysrhythmias), and elderly MDs combined with CVDs (E-MDs/CVDs) (e.g., hyperlipidemia plus hypertension). MDs and CVDs were diagnosed according to ICD-10 codes E00–90 and I00–99, respectively, by an experienced gerontologist who served in a medical center in northern Taiwan. The exclusion criteria included the following: patients without MDs and/or CVDs (e.g., hepatic disorders), those with malignant tumors and autoimmune diseases (e.g., rheumatoid arthritis), those who received an immunosuppressant, and those who were currently a hepatitis B or C carrier. Patients with other diseases were excluded. EH and YH controls were required to be physically healthy with no diagnosed pathological changes. The gender ratio and age range of each group are shown (Figure 2).

The relevant clinicodemographic parameters of participants are shown in Table 1. The study protocol was approved by the Ethics Committee of Chang Gung Medical Foundation (approval no.: 201700941B0C501, 15 August 2017). All participants signed an approved informed consent form containing the sentence, “If information from this study is published or presented at scientific meetings, your name and other personal information will not be shown.” All methods were performed according to relevant guidelines and regulations.

### 4.3. Methods

#### 4.3.1. Blood Sample Collection

PB samples (8 mL, with EDTA anticoagulants) were collected from each patient during routine outpatient tracking. Plasma was obtained from 2 mL of the sample through centrifugation at 1500 g for 10 min and then stored at −80 °C until analysis. The remaining fresh blood sample (6 mL) was used to determine immune cell subsets by performing a flow cytometric analysis (Attune^®^ NxT Acoustic Focusing Cytometer; ThermoFisher Scientific, Waltham, MA, USA) immediately after sample collection.

#### 4.3.2. Flow Cytometry and Antibodies

A cell suspension of PB from each patient was prepared according to standard protocols. Cell suspensions were stained for the flow cytometric analysis by using the following antibodies (obtained from BD Biosciences, San Jose, CA, USA unless otherwise stated): PerCP-Cy5.5-anti-human CD3; Alexa Fluor700-anti-human CD4; FITC-anti-human CD8; BV421-anti-human CD25; Alexa Fluro647-anti-human Foxp3; PE-anti-human Invariant NKT (iNKT; Vα24JαQ TCR chain); PerCP-Cy5.5-anti-human CD45RA; V450-anti-humanCD62L; APC-anti-human CD28; and PE-anti-human CD57.

For the analysis of iNKT and Treg cell populations, initially, PB mononuclear cells (PBMCs) (10^6^ cells/reaction) were stained with surface markers for CD3, CD4, CD8, CD25 and iNKT for 30 min at 4 °C in a dark room, and subsequently stained intracellularly for Foxp3 following the company protocol (BD Biosciences). Samples were then analyzed on an Attune^®^ NxT Acoustic Focusing Cytometer (ThermoFisher Scientific), and data were analyzed using Attune NxT software (ThermoFisher Scientific). The gating strategy for different immune cell populations is shown in Supplemental Figure S2. Isotype-matched immunoglobulins were used as controls.

Another staining reaction (PBMCs, 2 × 10^6^ cells/reaction) was undertaken for each patient, and samples were stained with surface markers for CD4, CD8, CD45RA, CD62L, CD28 and CD57 at 4 °C for 30 min. Lymphoid cells were gated for analysis of CD4, CD8 and subset profiles of both CD4^+^T cells and CD8^+^T cells, including T_N_ (CD45RA^+^CD62L^+^), central memory T (T_CM_; CD45RA^−^CD62L^+^), effector memory T (T_EM_; CD45RA^−^CD62L^−^) and effector memory T-cell re-expressing CD45RA T (T_EMRA_; CD45RA^+^CD62L^−^) populations. T_EM_ and T_EMRA_ populations of CD4^+^T cells and CD8^+^T cells were further subgated into four different subsets by using CD28 and CD57 markers, that is, T_EM_/T_EMRA_ with CD57^+^CD28^+^, CD57^+^CD28^−^, CD57^−^CD28^+^ and CD57^−^CD28^−^ populations. All data were acquired using an Attune^®^ NxT Acoustic Focusing Cytometer (ThermoFisher Scientific) and analyzed using Attune NxT software (ThermoFisher Scientific). The gating strategy of different immune cell populations is shown in Supplemental Figures S3 and S4. Isotype-matched immunoglobulins were used as controls.

#### 4.3.3. Quantitation of Serum IFN-γ, TGF-β, and GDF-15 Levels

IFN-γ, TGF-β and GDF-15 concentrations in human serum from each participant were measured using commercial enzyme-linked immunosorbent assay (ELISA) kits, namely IFN-γ and TGF-β ELISA kits (Invitrogen from ThermoFisher Scientific) and a GDF-15 ELISA kit (R&D System, Minneapolis, MN, USA) according to manufacturer’s instructions, and data were interpolated from the standard curves.

#### 4.3.4. Proliferation Assay

5-(and 6)-Carboxyfluorescein diacetate succinimidyl ester (CFSE) labeling was used to measure cell proliferation, as previously described [42]. Briefly, for each donor, 7 × 10^4^ CD3^+^ T-cells were cultured in RPMI 1640 medium supplemented with 10% fetal bovine serum (Gibco Life Technologies, Carlsbad, CA, USA) and stimulated with Dynabeads (anti-CD3/28; Gibco Life Technologies) and rIL-2 (30 U/mL) at a bead-to-cell ratio of 1:1. Cells were harvested following 2 and 4 days of culture and stained with the anti-PD1-PE antibody (BD Biosciences). Cell apoptosis was measured using an annexin V apoptosis detection kit (BD Biosciences), and beta-galactosidase (β-Gal) activity was detected using a Fluorometric assay kit (BioVision, Milpitas, CA, USA) according to manufacturer’s instructions. CFSE dilution was measured through flow cytometry by using an Attune^®^ NxT Acoustic Focusing Cytometer (ThermoFisher Scientific). Because the CFSE signal becomes diluted with each cell division, cells exhibiting a low CFSE fluorescence intensity were considered to have proliferated.

#### 4.3.5. Statistical Analysis

Data were analyzed using SPSS 13.0 software or GPower 3.1 software. All results are presented as the mean ± standard deviation. Descriptive statistics were used to determine whether results were normally distributed. One-way ANOVA with Bonferroni’s multiple comparison post-test was used for intergroup comparisons. Differences were considered significant at * *p* < 0.05, ** *p* < 0.01 and *** *p* < 0.001.

## 5. Conclusions

We observed alterations in T-cell subsets in elderly patients with MDs and CVDs. These patients tend to accumulate terminally differentiated T-cells in the circulation compared with healthy young or elderly controls. The elucidation of the association of elderly MDs and/or CVDs with the T-adaptive immune system and the mechanisms underlying these alterations could lead to biomarkers for disease prevention, diagnosis, or even therapeutic monitoring for these two common age-related diseases.

## Figures and Tables

**Table 1:** Demographics of participants.

Variables of Interest	YH (*n* = 19)	EH (*n* = 16)	E-MDs (*n* = 14)	E-CVDs (*n* = 14)	E-MDs/CVDs (*n* = 40)
Age (years), (mean ± SD)	31.2 ± 5.8	67.1 ± 5.7	68.2 ± 8.3	77.6 ± 11.7	71.2 ± 9.9
Sex (% female)	52.6	44	64.3	42.9	52.5
BMI (Male)	21.38 ± 4.58 ^a^	23.93 ± 4.79 ^b^	25.32 ± 2.71 ^c^	25.89 ± 1.94 ^d^	26.79 ± 2.17 ^e^
(Female)	22.09 ± 2.52 ^f^	22.27 ± 0.5 ^g^	25.29 ± 2.20 ^h^	25.08 ± 4.01 ^i^	27.38 ± 4.94 ^j^
WBC (10^9/L)	6.4 ± 1.6	5.5 ± 1.3 ^k^	6.4 ± 1.5 ^l^	6.5 ± 1.5 ^m^	6.3 ± 1.4 ^n^
Neutrophil (%)	59.0 ± 8.6	60.6 ± 9.1	55.1 ± 6.9	59.1 ± 10.5	58.1 ± 8.1
Monocyte (%)	6.0 ± 1.8	6.4 ± 2.0	6.1 ± 3.5	5.7 ± 1.3	5.8 ± 1.5
Lymphocyte (%)	31.3 ± 7.7	30.8 ± 9.1	35.6 ± 7.1	31.8 ± 9.8	33.1 ± 8
Diagnosis	no	no	Hyperlipidemia	COPD	Hypertension + hyperlipidemia
			Hyperuricemia	Hypertension,	Hypertension + hyperuricemia
			TypeII DM	Arrhythmia	TypeII DM + hypertension
					TypeII DM + hypertension + hyperlipidemia
					Hypertension + hyperlipidemia + CAD + asthma
					TypeII DM + hypertension + hyperlipidemia + coronary syndrome + CAD
					TypeII DM + hypertension + hyperlipidemia + hyperuricemia
Prescriptions	no	no	Statin	CCB	ARB + statin + Ezetimibe
			Statin + BI + DDP4I	β-blocker	CCB + ARB + Diuretic + statin + BI + DDP4I
			Ezetimibe + Fenofibrate	ARB	CCB + ARB + β-blocker + statin
			Vytorin	Diuretic + ARB	ARB + SU + BI
			Acarbose + BI + Pioglitazone	β-blocker + Diuretic + CCB + ARB	ARB + CCB + Diuretic
					ARB + Diuretic + CCB + β-blocker + statin + Febuxostat
					ARB + Diuretic + SU + Fenofibrate

* *p* < 0.05 (b to c and d); ** *p* < 0.01 (a to c.d, b to e, f to h.I, g to h.I, and k to l.m); *** *p* < 0.001 (a to e, f to j, g to j, and k to n). DM: Diabete Mellitus; CAD: Coronary Artery Disease; COPD: Chronic Obstructive Pulmonary Disease. CCB: Calcium channel blocker; ARB: Angiotensin II receptor blocker; BI: Biguanide; SU: Sulfonylurea; DDP4I: Dipeptidyl peptidase-4 inhibitor. YH, young healthy subjects; EH, elderly healthy subjects; E-MDs, elderly subjects with metabolic diseases; E-CVDs, elderly subjects with cardiovascular diseases; E-MDs/CVDs, elderly subjects with both MDs and CVDs; WBCs, white blood cells.

## Supplementary Materials

The following are available online at https://www.mdpi.com/2075-1729/10/10/246/s1, Figure S1: Comparison of WBCs counting in PB among different groups. Figure S2: Gating strategy of T lymphocyte subsets. Figure S3: Gating strategy of different subsets of CD8 T cells. Figure S4: Gating strategy of CD4 T cells. Figure S5: Comparison of T lymphocyte subsets in PB that affected by gender. Figure S6: Comparing blood-derived senescent CD4 T and CD8 T (CD28-CD57+) cells proliferation using anti-CD3/28 microbeads stimulation.
