# Supplementary material for: Alterations of Specific Lymphocytic Subsets with Aging and Age-Related Metabolic and Cardiovascular Diseases
Source: Life (Basel). 2020 Oct 17;10(10):246. doi: 10.3390/life10100246 (PMC7603042; doi:10.3390/life10100246)
Supplement: Supplementary file 1 [file life-10-00246-s001.pdf]

life

The supplementary materials of Alterations of Specific Lymphocytic Subsets with Aging and Age-Related Metabolic and Cardiovascular Diseases

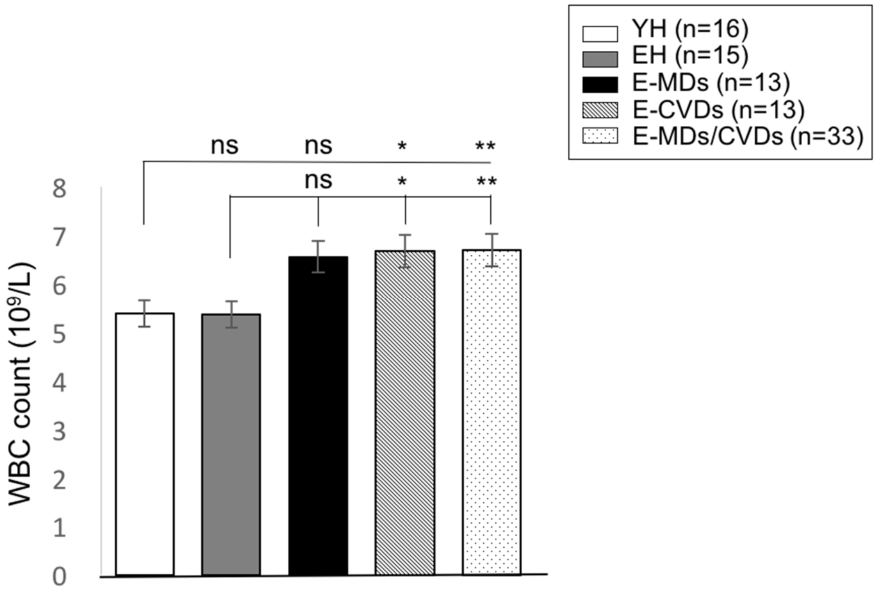

Figure S1. Comparison of white blood cells (WBCs) counting in peripheral blood (PB) between different elderly groups. Expression levels (10^9/L) of WBC cells were analyzed by regular CBC counting in PB from young healthy controls (YH, n=16), elderly healthy control (EH, n=15), elderly patients with metabolic diseases (E-MDs, n=13), elderly patients with cardiovascular diseases (E-CVDs, n=13), and elderly patients with both metabolic diseases plus cardiovascular diseases (E-MDs/CVDs, n=33). Data are shown as the mean ± SD of individual group comparison (*P < 0.05; **P < 0.01).

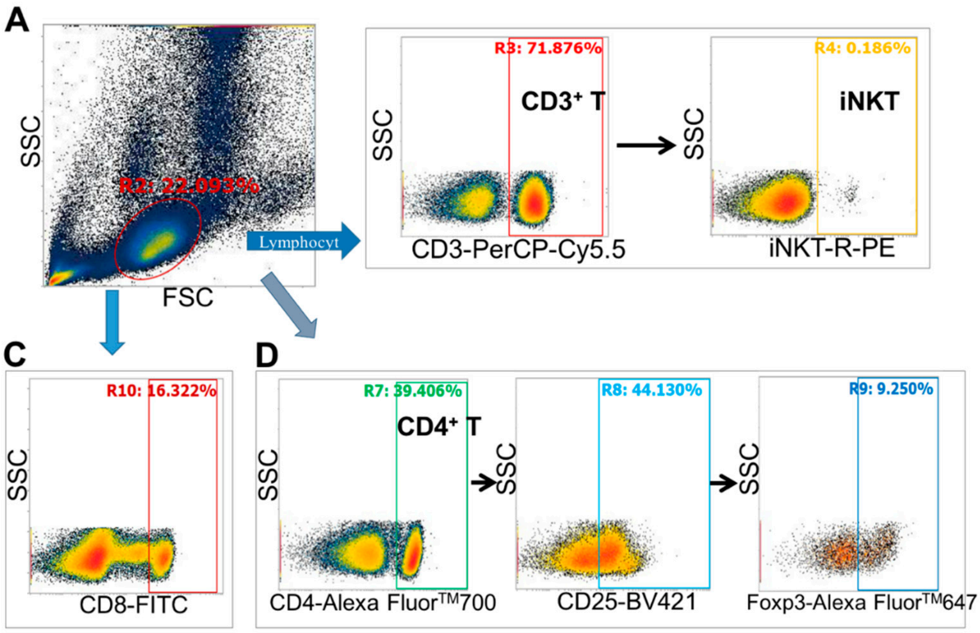

Figure S2. Gating strategy of T lymphocyte subsets. T lymphocyte subsets including cluster of differentiation 3 T (CD3T) cells, CD4T cells, invariant natural killer T (iNKT) cells, and regulatory T

(Treg, $CD4^+CD25^+Foxp3^{hi}$) cells were gated from a total of $5x10^5$ peripheral blood mononuclear cells (PBMCs)/collection using a flow cytometric analysis.

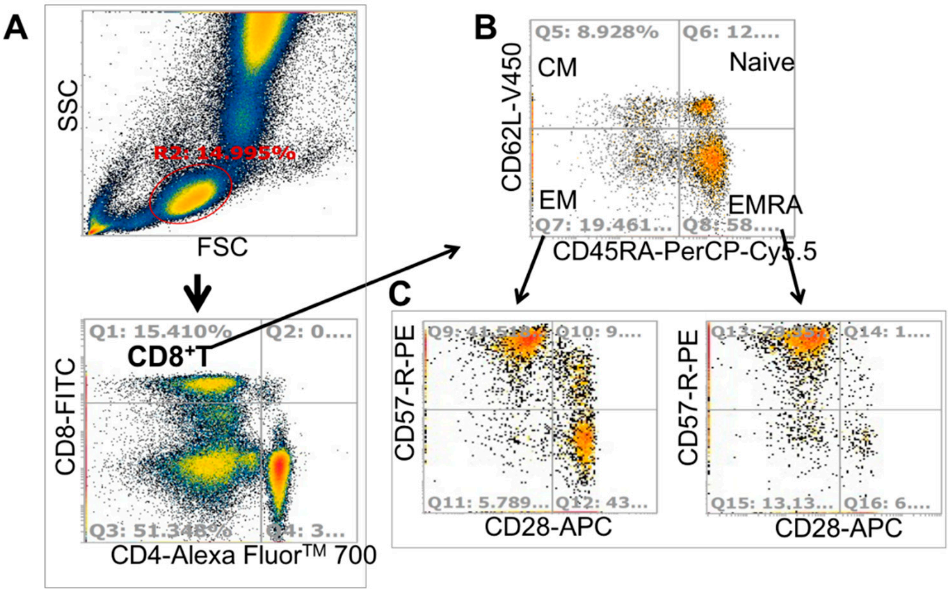

Figure S3. Gating strategy of different subsets of cluster of differentiation 8 T (CD8 T) cells. CD8T cell subpopulations, including naïve T (T_N, $CD62L^+CD45RA^+$), effector memory T (T_{EM}, $CD62L^-CD45RA^-$), effector memory re-expressing CD45RA T (T_{EMRA}, $CD62L^-CD45RA^+$), and the loss of CD28 and gain of CD57 ($CD28^-CD57^+$) T cell subsets under T_{EM}/T_{EMRA} were gated from a total of $5x10^5$ peripheral blood mononuclear cells (PBMCs)/each collection using a flow cytometric analysis.

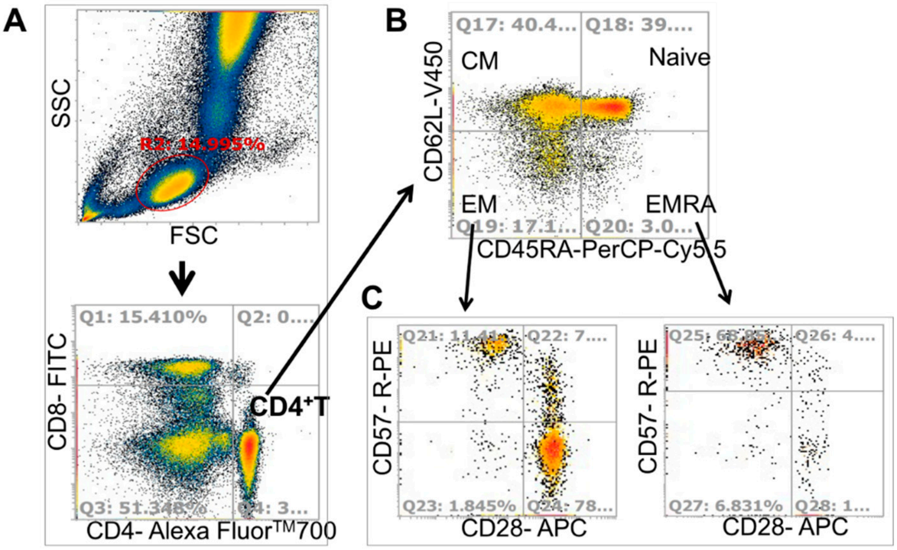

Figure S4. Gating strategy of different cluster of differential 4 T (CD4 T) cell subpopulations. CD4T cell subpopulations, including T_N, T_{EM}, T_{EMRA}, and the loss of CD28 and gain of CD57 ($CD28^-CD57^+$) fractions in T_{EM} and T_{EMRA} cells were gated from $5x10^5$ peripheral blood mononuclear cells (PBMCs)/collection using a flow cytometric analysis.

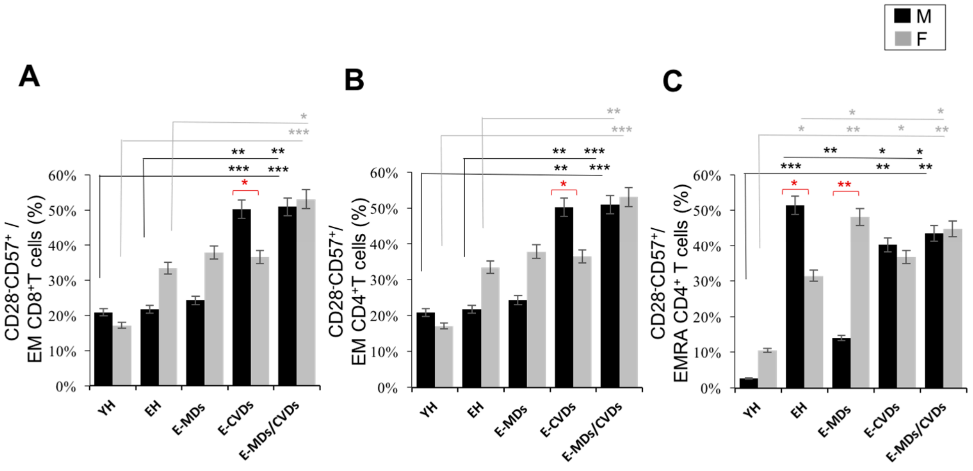

Figure S5. Comparison of T lymphocyte subsets in peripheral blood (PB) that affected by gender. Frequencies (%) of CD28-CD57+/CD8+T$_{EM}$ (**A**) and CD28-CD57+/CD4+T$_{EM}$ (**B**) subsets were significantly increased in male (M) with E-CVDs compared to female (F) with E-CVDs. The frequencies (%) of CD28-CD57+/CD4+T$_{EMRA}$ subset (**C**) was significantly increased in elderly male compared to elderly female, whereas it was increased significantly in female with E-MDs compared to male with E-MDs. Data analyzed by flow cytometry in PB from young healthy controls (YH, n = 11; M/F = 7/4), elderly healthy controls (EH, *n* = 11; M/F = 4/7), elderly patients with metabolic diseases (E-MDs, *n* = 12; M/F = 4/8), elderly patients with cardiovascular diseases (E-CVDs, *n* = 12; M/F = 7/5), and elderly patients with both MDs and CVDs (E-MDs/CVDs, *n* = 24; M/F = 13/11). Data are presented as the mean ± SD of individual group comparisons (**p* < 0.05; ***p* < 0.01; ****p* < 0.001). .

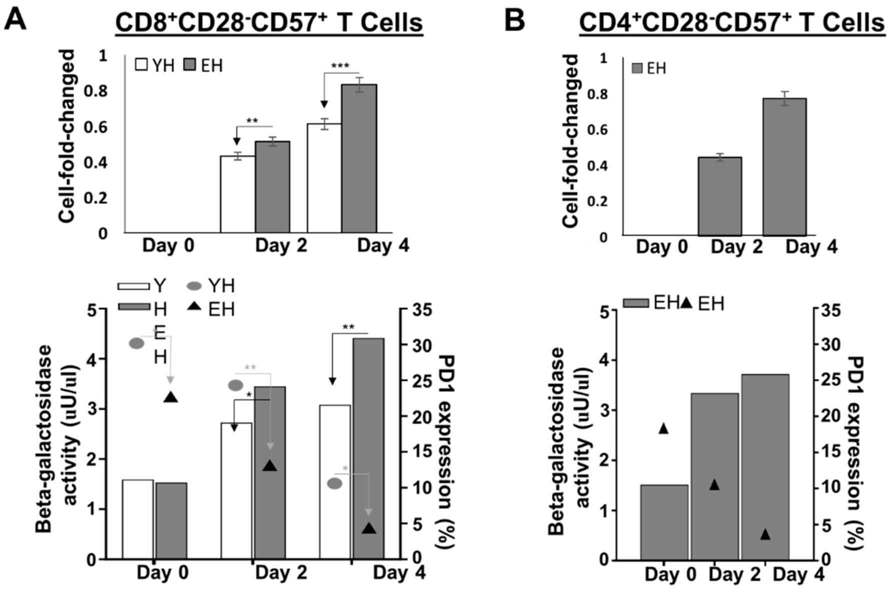

Figure S6. Comparing blood-derived senescent CD4 T and CD8 T (CD28$^-$CD57$^+$) cells proliferation using anti-CD3/28 microbeads stimulation. CD8$^+$CD28$^-$CD57$^+$ T and CD4$^+$CD28$^-$CD57$^+$ T cells from young healthy (YH) and elderly healthy (EH) (n=3 per group) were isolated from peripheral blood and cultured for 2 and 4 days following microbeads stimulation. (**A** and **B**, upper panel) Comparison of cells fold-changed after proliferation. (A and B, lower panel) Beta-galactosidase activity (*u*U/*u*l) and PD1 expression levels (%) comparison between YH and EH on days 2 and 4 in both senescent T cell groups. Data shown are the mean ± SD in each individual group comparison (**p* < 0.05; ***p* < 0.01; ****p* < 0.001).

© 2020 by the authors. Submitted for possible open access publication under the terms and conditions of the Creative Commons Attribution (CC BY) license (http://creativecommons.org/licenses/by/4.0/).
